# Characterization of metal(loid)s and antibiotic resistance in bacteria of human gut microbiota from chronic kidney disease subjects

**DOI:** 10.1186/s40659-022-00389-z

**Published:** 2022-06-17

**Authors:** María V. Miranda, Fernanda C. González, Osvaldo S. Paredes-Godoy, Mario A. Maulén, Claudio C. Vásquez†, Waldo A. Díaz-Vásquez

**Affiliations:** Molecular microbiology and food research laboratory, Facultad de ciencias para el cuidado de la salud, Escuela de nutrición y dietética, Universidad San Sebastián, Carmen Sylva 2444, Providencia Santiago, Santiago, Chile

## Abstract

**Background:**

Human Gut Microbiota (HGM) is composed of more than one thousand species, playing an important role in the health status of individuals. Dysbiosis (an HGM imbalance) is augmented as chronic kidney disease (CKD) progresses, as loss of kidney function accelerates. Increased antibiotic use in CKD subjects and consumption of nephrotoxic heavy metals and metalloids such as lead, cadmium, arsenic, and mercury in tap water increases the dysbiosis state. Studies in people with stage 3 CKD are complex to carry out, mainly because patients are self-reliant who rarely consult a specialist. The current work focused on this type of patient.

**Results:**

Lead and arsenic-resistant bacteria were obtained from self-reliant (that stands on its own) stage 3 CKD subjects. Pathogen-related Firmicutes and Proteobacteria genus bacteria were observed. Resistance and potentiation of antibiotic effects in the presence of metal(loid)s in vitro were found. Furthermore, the presence of the following genes markers for antibiotic and metal(loid) resistance were identified by qPCR: *oxa10*, *qnrB1*, *mphB*, *ermB*, *mefE1*, *arr2*, *sulll*, *tetA*, *floR*, *strB*, *dhfr1*, *acrB*, *cadA2k*, *cadA3k*, *arsC*, *pbrA*. We observed a decrease in the number of metal resistance markers.

**Conclusions:**

The presence of *cadA* and *arsC* genetic markers of antibiotics and metal(loid)s resistance were detected in samples from stage 3 CKD subjects. Lower gene amplification in advanced stages of CKD were also observed, possibly associated with a decrease in resident HGM during kidney disease progression.

**Supplementary Information:**

The online version contains supplementary material available at 10.1186/s40659-022-00389-z.

## Introduction

The human gut microbiota (HGM) is acquired rapidly after birth and evolves with age. A healthy intestinal tract and HGM are relatively stable throughout adulthood, but as a person ages, external elements such as antibiotic use, diet, and cellular stress, among others [1–3] trigger the so-called dysbiosis by altering HGM homeostasis. Specifically, antibiotic use influences HGM composition by eliminating sensitive bacteria and generating selection pressure, which influences the multi-resistance antibiotic ability of microorganisms [4].

In terms of bacterial species, between 1000 and 1150, mostly uncultivable, species have been described in healthy adults. The HGM is composed of 6 predominant phyla: Firmicutes, Bacteroidetes, Actinobacteria, Proteobacteria, Fusobacteria, and Verrucomicrobia; with Firmicutes and Bacteroidetes corresponding to 90% of the intestinal bacterial population [5].

Although carbohydrates are the preferred substrates for fermentation in the proximal colon, proteins may also be used as an energy source [6]. Amino acid degradation by the HGM occurs by oxidation deamination, producing NH_3_, short-chain fatty acids (SCFA), hydrogen sulfide, and metabolites including phenol, p-cresol, indol, and scatol derivative compounds [7]. These compounds, in turn, produce microbial-derived uremic toxins that affect the progression of chronic non-communicable diseases, such as chronic kidney disease (CKD) [8, 9] and cardiovascular disease [10]. Firmicutes and Bacteroidetes participate in SCFA synthesis, reducing the inflammatory state and immune responses [11].

Chronic Kidney Disease (CKD) affects approximately 10% of the world population [12]. The progression is defined as the loss of function maintained for ≥ 3 months, expressed by a glomerular filtration rate (GFR) < 60 mL/min/1.73 m^2^. [13]. CKD is closely associated with cardiovascular disease; cardiovascular mortality can be up to 15 times higher in CKD patients compared to the general population [14]. Stage 3 CKD (CKD3) is particularly complex to manage, mainly because patients are self-reliant and rarely consult a nephrology specialist, therefore the number of CKD3 patients is unknown.

Daily exposure to arsenic, cadmium, mercury, and lead in tap water raises the possibility of increased microorganism resistance or tolerance to these toxicants [15]. The presence of metals also contributes to increased antibiotic resistance through co-selection. Co-selection can occur due to co-resistance phenomena, where 2 or more resistance genes are in the same genetic element and regulated by the same promoter, or cross-resistance, where a single resistance mechanism confers resistance to different components simultaneously, such as the cadmium efflux pump that can expel beta-lactam antibiotics [16]. These issues may be particularly important for people with CKD compared to healthy subjects. In Chile, maximum concentrations for metals permitted in tap water is defined by Chilean standard NCH 409/1 (0.01 mg/L arsenic, 0.01 mg/L cadmium, 0.001 mg/L mercury and 0.05 mg/L lead) [17].

Recently, profiles of antibiotic resistome and the microbial community in groundwater have been analyzed in areas where CKD of unknown etiology (CKDu) appear. Researchers have shown a positive correlation between CKDu, resistome, and microbial communities found in drinking water [18]. The presence of CKDu in the population has also been associated, among other factors, with consumption of cadmium, arsenic, lead, and aluminum [19].

In this work, we explored the interaction of metal(oids) and antibiotics in CKD patients, which would reflect both metal(loid)s and antibiotic resistance and influences the number of genetic determinants associated with the metabolism of antibiotics and metal(loid)s present in microbiota. Our main goal was to analyze changes on HGM over CKD course and to characterize the resistance to metal(loid)s and antibiotics of microorganisms obtained from fecal samples. We tested whether there were changes in resistance in the different stages of disease progression. To our knowledge, this is the first work exploring the relationship between daily exposure to metal(loid)s and the presence of bacterial genetic markers of resistance to metal(loid)s and/or antibiotics in patients. We hypothesized that there would be a higher number of resistant microorganisms and genetic markers associated with metal(loid)s and antibiotics resistance, and this number increase over the course of CKD, we infer that the change would be related to the augmented presence of these toxicant substances because of kidney failure and increased antibiotic due to recurrent infections in CKD patients.

## Materials and methods

Sixteen patients participated in this study, with 4 individuals per group: stage 3, stage 4, stage 5 and healthy controls. To select study subjects, clinical records and previous laboratory tests were reviewed. We selected patients who had values of glomerular filtration rate (eGFR) that allowed for stage classification based on serum creatinine concentration. Classification of subjects was defined between 30 and 60 mL/min/1.73 m^2^ of eGFR (KDIGO). We followed guidelines for defining CKD including (a) structural or functional kidney abnormalities persisting over 3 months; (b) persistent proteinuria including microalbuminuria (MAU), A1-A2 categories < 300 mg/g; and (c) decreased filtration rate (less than 60 mL/min per 1.73 m^2^ [20]. The inclusion criteria included healthy controls and CKD subjects between 30 and 65 years of age. Kidney replacement therapy (hemodialysis or peritoneal dialysis) only among stage 5 CKD subjects. The exclusion criteria included persons with a history of chronic digestive and intestinal pathologies and/or malabsorption syndrome, autoimmune or chronic inflammatory disease, or cancer. Drug consumption including tobacco, alcohol, antibiotics (< 6 months of non-consumption), anti-inflammatories, and laxatives, except those prescribed for treatment of CKD and pregnancy were considered exclusion criteria. The sample size was calculated using the G*power 3.1 software considering study power of 0.95 and 0.05 error, using the effect size of 75%.

After reviewing the protocol, participants signed a consent form. All documents and protocols were reviewed and approved by the scientific ethics committee of the Eastern Metropolitan Health Service and San Sebastián University.

### Nutritional and biochemical parameters

Nutritional parameters were measured by a nutritionist. Height and weight were measured using a stadiometer (Seca 217) and scale (Seca 876), respectively, and BMI calculated. The waist and hip circumference were measured using the tape measure and waist to hip ratio calculated. Bioimpedanciometry was performed using the XCONTACT 350 device and percent of lean body mass was measured (D1000-3). The following biochemical parameters were analyzed by an external laboratory: albuminuria (mg/24 h), creatinine (mg/L), urea (g/L), glucose (mg/dL), albumin (g/dL), calcium (mg/dL), and phosphorous (mg/dL) (Additional file 5: Table S1).

### Collection and preservation of samples

Stool samples were collected separately according to group (stage 3, 4, or 5 or healthy controls). Samples were processed in class II biosafety cabinets, aliquoted in 1.5 mL tubes, and stored at − 20 °C until processing.

### *E. coli* isolation protocol and determination of the minimum inhibitory concentration (MIC) of metal(loid)s

In 200 mg of previously aliquoted fecal samples, 1 mL of YCFAm [21–23] was added to into a 1.5 mL tube. The mix was homogenized by pipetting and samples centrifuged for 30 s at 13,000×*g*. The resulting supernatant was used for bacterial isolation procedures, growth curves, resistance/tolerance to metal(loid)s and antibiotics resistance assay.

Two hundred μL of the mix was spread in Eosin methylene blue Levine agar to grow at 37 °C for 16 h. Bacteria that showed a metallic green coloration was isolated in YCFAm medium, the colonies were used in the minimum inhibitory concentration (MIC) assays. 20 mM of Mercury (Hg), arsenic (As) and lead (Pb), and 50 mM cadmium (Cd) stock solution was used for preparing tenfold serial dilutions. Later, 10 µL of a saturated culture of bacteria was inoculated into each dilution under aerobic conditions. Because *E. coli* is considered a sensitive bacterium for metal(loid)s toxicant effects, the MIC of *E. coli* was used as a cut-off point to determine resistant/tolerant microorganisms for metal(loid)s in stool samples.

### Determination of resistant microorganisms

Plates with 2% agar YCFAm plus *E. coli* MIC of lead and arsenic were used to seed 100 µL of stool samples and select resistant/tolerant for further experiment. Colonies that grew in the presence of each of the metal(loid)s analyzed were stored in 30% glycerol at − 80 °C. The number of colony-forming units per gram (CFU/g) was determined after incubating at 37 °C for 24 h. The count of viable cells was performed by Image pixel analysis in ImageJ 1.8 software.

### Determination of antibiotic resistance in bacteria

Microorganisms that showed resistance/tolerance to indicated metal(loid)s were analyzed using the Kirby Bauer method [24]. Antibiotic paper disks of gentamicin (10 µg), ampicillin (10 µg), ciprofloxacin (5 µg), cefazolin (30 µg), ceftazidime (30 µg), and gentamicin (120 µg) were placed on bacteria previously seeded on agar plates in presence of indicated metal(loid)s and incubated at 37 °C for 24 h. Growth inhibition zones were measured and compared with the standardized diameter by The Standards for Antimicrobial Susceptibility Testing (CLSI) [25].

### Growth curves

Bacteria grown for 16 h in culture were used to take inoculum and diluted by 1:1000 in fresh YCFAm broth and cultivated. When necessary, the medium was supplemented with ½ MIC of *E. coli* of each metal(oid) toxicant tested in 48-well plates (SPL life sciences) at 37 °C for 18 h with orbital shaking. The dynamics of grown bacteria were estimated by optical density at 600_nm_.

### DNA isolation procedure

Stool samples were thawed at 4 °C and 200 mg of sample was aliquoted in a 1.5 mL tube containing 200 mg of beads for mechanical disruption. TE buffer was added for resuspension, a vortex was applied at 2500 rpm for 10 min, then the sample was centrifuged for 1 min at maximum speed and transferred to a clean tube. Proteinase K was added to a final concentration of 1 mg/mL and incubated at 50 °C for 10 min. Later stages were performed according to manufacturer instructions (Power Fecal kit protocol Qiagen) and, finally, the eluted DNA was stored at – 20 °C until use.

### 16S rRNA amplicon sequencing

16S rRNA V4 region sequencing was carried out using Miseq sequencing (Illumina). Primers 515F (5′-GTGCCAGCMGCCGCGGTAA-3′) and 806R (5′-GGACTACHVGGGTWTCTAAT-3′) were used to generate a product of 250 bp with a high ratio between the observed and predicted communities (R^2^ = 0.95) [26]. Taxonomic characterization of bacterial populations present in YCFAm colonies able to grow in metal(oid)s plates from the microbiota of CKD3 patients was performed by phylogenetic analysis on Rstudio (v3.53) [27]. The quality of the sequence obtained by forward and reverse primers was determined using the DADA2 (v1.12.1) [28] package and the SILVA database [29] for phylogenetic assignation. Only operational taxonomic units (OTUs) that obtained a relative abundance > 0.2% of total readings and > 1000 readings per taxa were considered.

### Selection and synthesis of primers for genetic determinant analysis

**Fig. 1:**
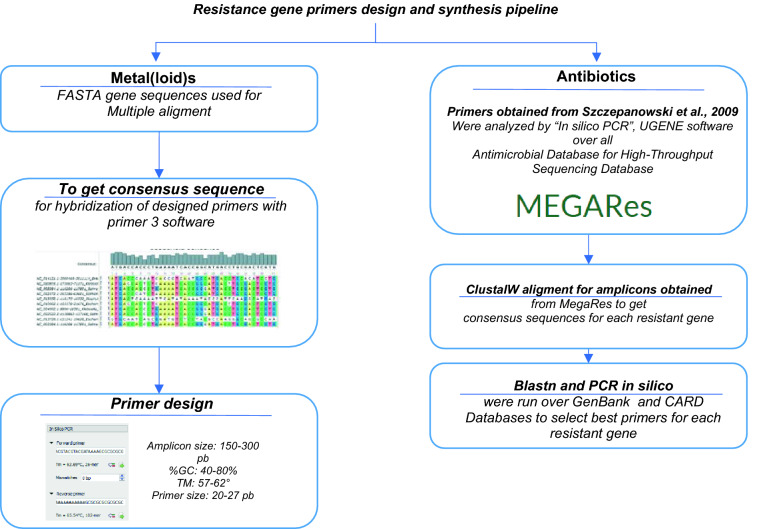
Selection and synthesis of primers for genetic determinants analysis. Primers were used to amplify resistance genes to metals and antibiotics in DNA isolated from stool samples. For antibiotics analysis, primers were obtained from the literature [30], later an alignment was carried out with all databases of MEGARes [31], CARD [32], and GenBank [33] and evaluated by “in silico” PCR from UGENE [34]. For metal(loid) resistance primer design, principal antibiotic resistance genes belonging to each 6 phyla of HGM were aligned to determine the consensus sequence. Primers were designed with Primer3 software [35, 36]

Primers that amplify antibiotic resistance genes were obtained from the literature [30] and an alignment was carried out to find main hits of these oligos in the MEGARes [31], CARD [32] and GenBank [33] databases. Selected primers were subsequently evaluated by “in silico” PCR using UGENE software (Unipro) [34] (Fig. 1). For metal(loid) resistance primers design, principal resistance genes belonging to each of the 6 phyla of HGM were aligned to determine the consensus sequence. Sequence primers were designed with Primer3 software [35, 36]. Software conditions included: amplicon between 150 and 300 bp, GC percentage from 40 to 80%, Tm between 57 and 62 °C, and starter size between 20 and 27 bp. Selected primers were analyzed with “in silico” PCR (Fig. 1). The list of end primers used in this work is provided in the supplementary material (Additional file 6: Table S2).

### qPCR conditions

Total DNA was analyzed by real-time PCR (qPCR), using a bright green 2 × qPCR Mastermix PCR without ROX (ABM). PCR was performed using Quantstudio 3 equipment and melting curves were analyzed for each amplicon to ensure the presence of a unique product of the expected size.

### Statistical analysis

Statistical analysis was performed using GraphPad prism V9.2 software, except those corresponding to genomic analysis, which was performed using ggplot and analyzed on Rstudio V 3.51. We used the ANOVA test, considering the significance of P < 0.05 (*), P < 0.01 (**) or P < 0.001 (***).

## Results and discussion

In this work we carried out exploratory research regarding the presence of microorganisms resistant to meta(oid)s and antibiotics in patients with CKD. We also performed an analysis of total DNA of stool samples to find resistance determinants in each patient, depending on CKD stage.

To analyze resistance/tolerance of metal(loid) and resistance to antibiotics in bacteria, samples from patients with CKD were analyzed in YCFAm culture medium using the MIC calculated from *E. coli* isolated from fecal samples (Additional file 7: Table S3). Our results exhibited colony growth in the presence of arsenic and lead under aerobic conditions (Additional file 1: Figure S1). No colonies were found on plates containing YCFAm medium in the presence of Hg and Cd (data not shown). Colonies harvested from each plate were used to perform an antibiotic resistance analysis.

**Fig. 2:**
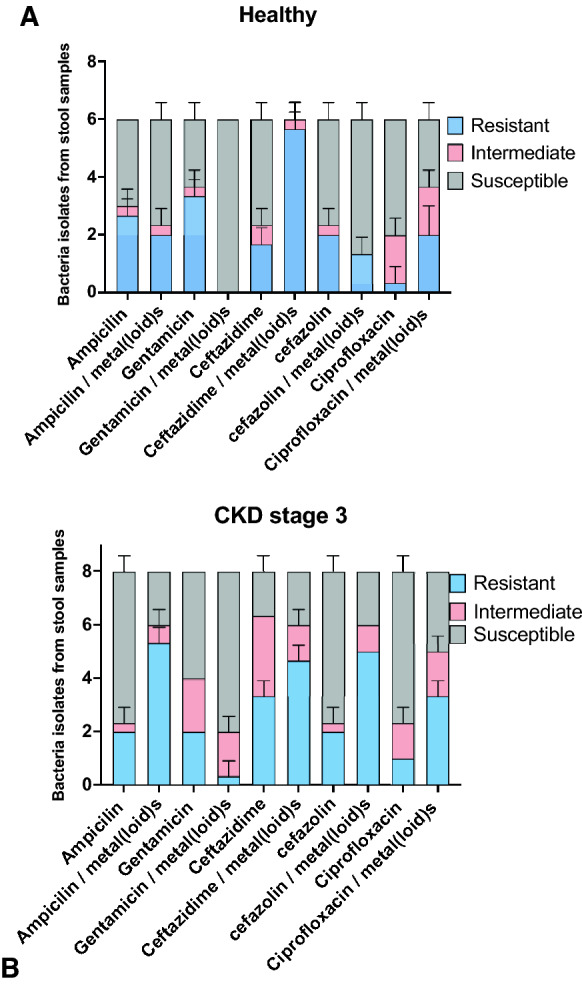
Susceptibility to antibiotics in bacteria isolated from stool samples of healthy patients and those with stage 3 chronic kidney disease. Inhibitory zones of bacteria isolated from stool samples were plated on YCFAm agar using the Kirby Bauer method [24]. Paper disks containing gentamicin (10 µg), ampicillin (10 µg), ciprofloxacin (5 µg), cefazolin (30 µg), ceftazidime (30 µg), and gentamicin (120 µg) were placed on previously seeded bacteria on agar plates and incubated at 37 °C for 24 h. Growth inhibition zones were measured and compared with the standardized diameter described by CLSI [25]. ½ MIC of arsenic and lead of *E. coli* was used to evaluate the effect of metal(loid)s on antibiotic resistance, n = 12. **A** healthy subjects, **B** CKD Stage 3 subjects

Bacterial isolates were analyzed using the Kirby Bauer method in the presence of paper disks with antibiotics and characterized according to CLSI parameters for resistant, intermediate, or sensitive bacteria (Fig. 2A and B) in the presence or absence of ½ MIC of metal(oid)s, a sublethal concentration. For healthy subjects, a decreased appearance of gentamicin resistance in the presence of metal(oid)s, ceftazidime, and ciprofloxacin was observed compared with healthy subjects, which indicates a significant increase in resistance to antibiotics in the presence of metal(loid)s. For CKD3 patients, no significant difference in antibiotic resistance was observed compared with the healthy controls and ½ MIC of *E. coli* for metal(loid)s (arsenic and mercury both). However, higher numbers of microorganisms resistant to antibiotics ampicillin, cefazolin, and ciprofloxacin were shown in plates containing ½ MIC of *E. coli* metal(loid)s. No differences between groups (CKD3 versus healthy controls) and toxicant presence were observed when growth curves of stool samples in the presence of ½ MIC of metal(loid)s (Additional file 2: Figure S2).

**Fig. 3:**
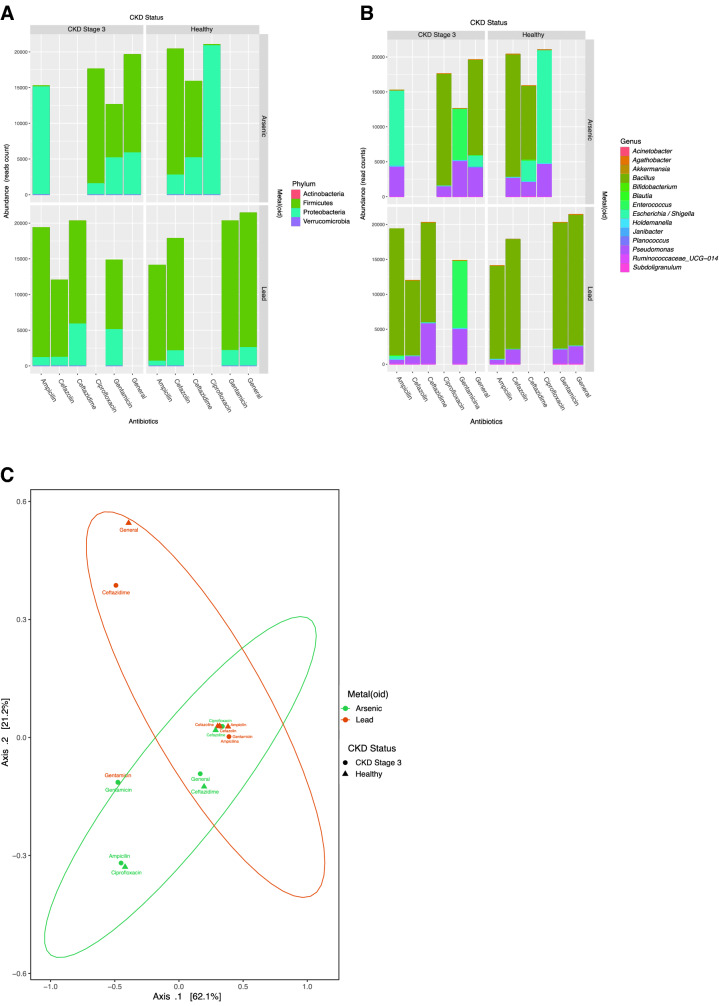
Relative abundance of bacteria. Taxonomic profiling of the 16 s rRNA amplicon was performed from colonies that showed antibiotic resistance. Colonies were isolated from healthy and stage 3 CKD subjects and compared in the presence of arsenic, lead, and antibiotics (ampicillin, cefazolin, ceftazidime, ciprofloxacin, and gentamicin) with general or multi-resistance. The abundance of each sample was performed by read count analysis of the phylum (**A**) and genus (**B**). **C** beta-diversity was determined by principal component analysis (PCA) and results sorted by OTUs > 0.2%. Experimental groups are indicated by color and shape, arsenic (green) and lead (orange), healthy subject (triangle) or CKD patients (circle)

To identify microorganisms in fecal samples that showed co-resistance to antibiotics and metal(loid)s, sequences of isolated colonies were analyzed by 16S amplicon sequencing. All isolates of healthy subjects and CKD subjects from each disease stage (3, 4, and 5) were compared after antibiotic treatments in the presence of ½ MIC of *E. coli* of arsenic or lead. For CKD3 patients and lead treatment, Firmicutes and Proteobacteria were mainly found (Fig. 3A). Genus analysis showed *Pseudomonas* spp., *Janibacter* spp., *Escherichia*/*Shigella* spp., and *Bacillus* spp.; resistant bacteria to gentamicin, cefazolin, ceftazidime, and ampicillin were also found. For CKD3 patients and arsenic treatment *Pseudomonas* spp., *Escherichia* spp./*Shigella* spp., *Bacillus* spp. were observed; and *Enterococcus* spp. showed resistance to ampicillin, ciprofloxacin, and gentamicin. Furthermore, some colonies of interest exhibited resistance to all tested antibiotics (general resistance). On the other hand, for healthy controls and lead treatment, we observed mainly *Bacillus* spp. and *Pseudomonas* spp. and resistance to ampicillin, cefazolin, gentamicin, and colonies with a general resistance. Healthy controls and arsenic treatment exhibited Bacillus spp., *Escherichia* spp./*Shigella* spp. and *Pseudomonas* spp. (Fig. 3A–C). It was impossible to detect microorganisms of Phylum Bacteroidetes, mainly because of other selection phenomena such as the culture medium used (YCFAm) and aerobic conditions. Parameters of microorganism abundance in samples grown in the synthetic and selective media were performed, Chao1, Shannon, and Simpson indices were calculated [S2A for metal(loid)s and S2B for antibiotics]. Alpha diversity indexes were associated with the selection pressures presented in healthy patients and CKD3, although, these parameters were not analyzed in depth.

We found that the *Bacillus* spp. in the presence of metal(loid)s. showed multiple resistance to ampicillin, gentamicin, cefazolin, ceftazidime and ciprofloxacin *Pseudomonas* spp. showed the same behavior in the presence of metal(loid)s, exhibiting the same antibiotic resistance, but less representation.

On the other hand, *Enterococcus* spp. and *Escherichia* spp./*Shigella* spp. exhibited resistance to ampicillin and gentamicin, respectively. We noted that both healthy controls and CKD3 patients showed some bacteria with general resistance to antibiotics, but resistance differed mainly in the type of metal (arsenic for CKD stage 3 and lead for a healthy group). Most studies that have found this co-resistance phenomena in stool samples have been conducted in wastewater systems [37]. The phenomenon of co-selection allows conserving and promoting resistance to antibiotics and resistance/tolerance of metal(loid)s in bacteria, even in the absence of antibiotics through different co-resistance mechanisms, which opens the way to future investigations [17].

In taxonomic profiling, we identified the presence of *Janibacter* spp., which has been recently reported to be involved in the virulence process related to antibiotic resistance in HGM of celiac and immunocompromised subjects [38], expressing proteins such as β-lactamase and other 7 genes related to metal(loid) resistance/tolerance including arsenic [39]. This led us to perform further analysis to characterize the isolated strain and identify the genes when this gut pathogen was exposed to metal(loid)s and antibiotics.

Beta diversity analysis between control and CKD stage 3 isolated bacteria showed no significant differences when stool samples were cultured under aerobic conditions and when exposed to metalloids and different antibiotics (Fig. 3C). This could be explained by a continuous process of the multi-resistance capacity of bacteria. Interestingly, this result suggests that the early stages of CKD do not influence the development of multi-resistant bacteria. To explore this idea in depth, we performed an analysis to detect resistance markers by qPCR.

It was initially suggested that CKD patients have augmented resistance genes in HGM because of a possible accumulation or transcendent increase of toxicant compounds such as metal(loid)s related to decreased renal filtration rate and the use of antibiotics for disease treatment. To determine the presence of resistance genes and genetic determinants related to metal(loid)s and antibiotics resistance in stool samples of healthy patients and to compare with subjects at different stages of CKD (3, 4, and 5), qPCR was performed (Additional file 6: Table S2). Primers were constructed to amplify regions of selected genes for metal(loid) resistance analysis corresponding to arsenic (*arsC*, *arsA*), lead (*pbrA*), mercury (*merA*), and cadmium (*cadA*). Specifically, for cadmium, 2 primers *cadA2k* and *cadA3k* were designed because in silico PCR results against MEGARes and CARD databases showed amplicons of different sizes and quantities. To increase coverage, both primers were synthesized.

To choose primers for antibiotic resistance genes, 140 described genes were analyzed [30] and 13 genes that represented each family were selected. For genes such as *floR*, a gene product resistance to chloramphenicol, a second product was initially detected in melting curves (not shown). To correct this bias, qPCR was performed with an annealing temperature gradient (60, 65, and 70 °C). It was observed that at 65 °C, only a single specific product was obtained. *sulll* primers of Sulfonamides dihydropteroate synthetase described by the group of Szczepanowski et al. [30] could not be used due to the detection of nonspecific products when analyzing melting curves in our samples (not shown). This could mean nonspecific interactions in other DNA regions, and therefore nonspecific amplification in our samples, producing false-positive results.

**Fig. 4:**
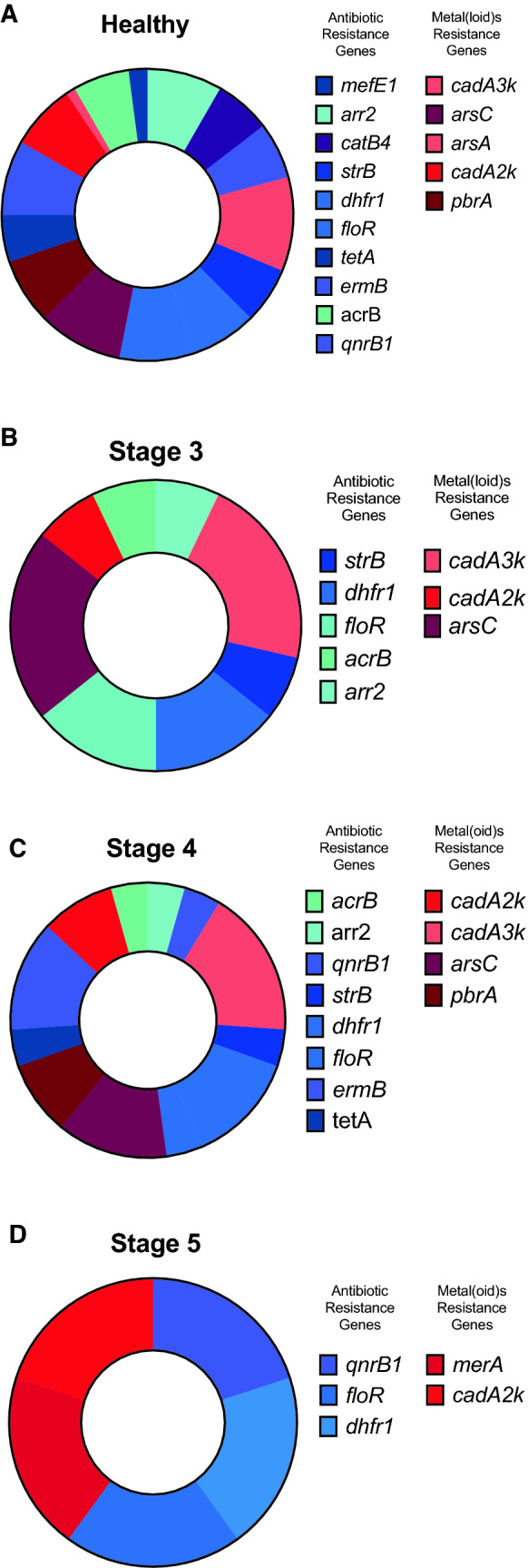
Pie charts indicating antibiotic resistance genes (cold colors) and metal(loid)s (warm colors) analyzed by qPCR of DNA obtained from stool samples. **A** *mefE1*, *arr2*, *catB4*, *strB*, *dhfr1*, *floR*, *tetA*, *ermB*, *acrB*, *qnrB1*, *cadA3k*, *arsC*, *arsA*, *cadA2k*, and *pbrA* genes were detected in healthy controls. **B** *strB*, *dhfr1*, *floR*, *acrB*, *arr2*, *cadA3k*, *cadA2k,* and *arsC* genes were detected in subjects with stage 3 CKD. **C** *acrB*, *arr2*, *qnrB1*, *strB*, *dhfr1*, *floR*, *ermB*, *tetA*, *cadA2k*, *cadA3k*, *arsC,* and *pbrA* genes were detected in subjects with stage 4 CKD. **D** *qnrB1*, *floR*, *dhfr1*, *merA,* and *cadA2k* genes were detected in subjects with stage 5 CKD. The graph represents the total appearance of resistance genes in 4 patient samples analyzed by group. The total identified appearances were 96 hits for healthy subjects, 14 hits for stage 3 CKD, 23 hits for stage 4 CKD and 5 hits for stage 5 CKD. CKD groups were compared with healthy controls using Two-Way ANOVA with Dunnett’s test for multiple comparisons (two-tailed) P < 0.0001 (****)

Pie charts (Fig. 4) describe the presence of antibiotic and metal(loid) resistance genes. Interestingly, the healthy control group (Fig. 4A) showed a higher number of resistance markers of metal(loid)s and antibiotics. In the same group, all analyzed genes for resistance to antibiotics and metals were found. The lack of some markers could not be evidenced in this group in comparison with the different stages of CKD. From CKD stage 3 samples (Fig. 4B), the presence of *strB*, *dhfr1*, *floR*, *acrB*, and *arr2* for antibiotic resistance genes and *cadA3k*, *arsC,* and *cadA2k* for metal(loid)s resistance genes were observed. In the same group *mefE1*, *catB4 and qnrB1* were not found. For stage 4 samples (Fig. 4C), the presence of *acrB*, *arr2*, *qnfrB1*, *strB*, *dhfr1*, *floR*, *ermB,* and *tetA* antibiotics were found and *cadA3k*, *arsC*, *cadA2k,* and *pbrA* for metal(loid) resistance genes, *ermB* was found again in stage 4. Concerning metal resistance genes, *arsA* and *pbrA* were not observed in CK3 subjects, however *pbrA* was identified in stage 4 patients. For stage 5 CKD samples (Fig. 4D), which correspond to subjects who no longer have renal function and require kidney replacement therapies, such as hemodialysis or peritoneodialysis and Firmicutes versus Bacteroidetes ratio tends to decrease because of the uremic state and increased proteolytic metabolism [7, 8], both groups of genes, for resistance to antibiotics and metal(loid)s were diminished compared with healthy subjects. The presence of *qnrB1*, *dhfr1,* and *floR* genes for resistance to antibiotics and *cadA2k* and *merA* for metal resistance was observed. We consider that the typical state of intestinal microbiota dysbiosis associated with CKD produces changes in microbiota that are associated with a smaller number of genetic determinants.

The study of the presence of metal(loid) resistance genes and antibiotics from the early stages of the disease is important because, at that stage, there are alterations in HGM composition -or dysbiosis- that could explain the decrease in the appearance of the analyzed genetic markers. Other alterations associated with the progression of disease include intestinal transit alterations, decreased protein absorption, decreased consumption of dietary fiber, and frequent use of antibiotics. These accumulative factors will contribute to the production of systemic inflammation and the bioaccumulation of uremic toxins and presumably metal(loid)s, which are absorbed by the intestine and eliminated by the kidney, these toxicants may play a central role in the physiopathology of CKD [40].

**Fig. 5:**
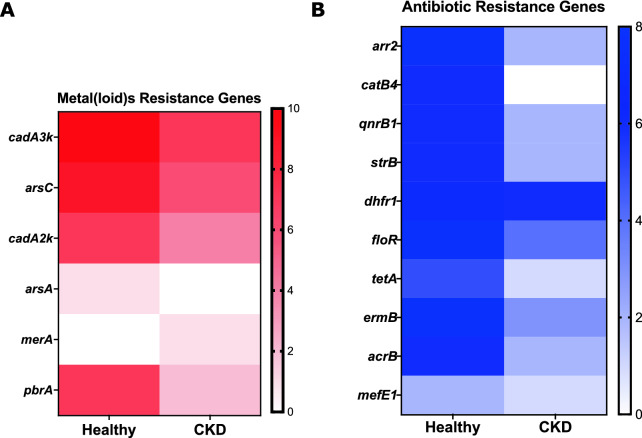
heat maps showing representative metal(loid)s resistance and antibiotic genes detected in samples from healthy versus CKD (all stages) subjects. **A** metal(loid)s resistance genes: *cadA3k*, *arsC*, *cadA2k*, *arsA*, *merA* and *pbrA*. For all cases analyzed, there was a tendency exhibit lower number of resistance genes to metal(loid)s, in particular, CKD patients tended to present a higher amount of *cadA3k* and then *arsC* compared to other resistance markers. In healthy patients, there was a tendency to present *arsC* and then *cadA2k*. **B** Antibiotic resistance genes observed were: *arr2*, *catB4*, *qnrB1*, *strB*, *dhfr1*, *floR*, *tetA*, *ermB*, *acrB,* and *mefE1*. The augmented tendency was observed for the *dhfr1* gene in patients with CKD. Healthy patients tended to present *arr2* and *ermB* genes, in slightly higher amounts compared to CKD patients. Groups were compared using Student t test, P < 0.0001 (***)

A high presence of resistance genes for cadmium and arsenic metabolism were observed in analyzed samples (Fig. 5A). Cadmium (Cd) is a heavy metal, usually present in soils [41]. It is toxic to living organisms, is carcinogenic to humans [41–44], and lethal [43]. The human body can absorb Cd in small portions through food intake, especially in grains, water, or air [43], where it accumulates and remains for a long time, causing health problems [43–47]. Cd accumulates in the liver and kidneys and has a long biological half-life, 17–30 years in humans. Toxicity involves 2 organ systems, the renal and skeletal, and is largely the consequence of the interactions between Cd and essential metals, particularly calcium [43, 45, 47]. The severity and damage of these metals depend on time, level of exposure, and susceptibility of the person, and which metal is absorbed [43].

Mining activities in the second region of Chile represents one of the main commercial and labor activities of the country. This industry emits waste rich in arsenic and other minerals, which affects both the environment and residents of the area [48, 49]. Arsenic is found food, water, and air. The main damage produced by arsenic exposure is cardiovascular, kidney, neurological, respiratory, skin cancer, and reproductive effects [50].

High levels of the *dhfr1* gene (dihydrofolate reductase) (Fig. 5B) can be observed on the heat diagram for both the healthy and CKD group, which corresponds to a resistance gene present in some *Escherichia coli* strains (https://www.uniprot.org/uniprot/Q0MQM2), the main causative microorganism of urinary tract infection (UTI) in healthy and CKD population [51].

To determine whether the observed changes in the content of antibiotic and metal resistance genes were significant between groups, a correlation (Pearson R) of Contingency of Prospective data (chi-square test) was performed and plotted by heat map (Additional file 4: Figure S4A) and principal component analysis PCA (Additional file 4: Figure S4B). Our results showed that healthy subjects (control), CKD3, and stage 4 CKD subjects are considered similar groups with a positive correlation. On the contrary, the stage 5 CKD group did not show a correlation with the previous groups. The results show that the gene content for resistance to antibiotics and metals is substantially lower than in the previous groups, Thus, further studies are needed to determine how late stages of CKD relate to a decrease in resistance markers.

The incidence of UTI in CKD patients increases with disease progression and occurs mainly due to the presence of risk factors. For example, uremia causes alterations in the humoral response, lymphocyte function, macrophages, and polymorphonuclear cells [51–53].

The underlying symptom of CKD is sometimes a condition that compromises normal elimination of urine and integrity of the urinary tract or implies its manipulation due to medical procedures (e.g., vesicoureteral reflux, neurogenic bladder, urethral valves, prostatism, bladder catheterization, renal catheterization, complicated lithiasis, and polycystic disease). In other cases, diabetes is an underlying symptom of both CKD and greater susceptibility to the appearance of UTI and its evolution and occurs especially in elderly female patients [51].

This study represents a slightly closer look at the association between the microorganisms present in the intestine and the evolution of a chronic disease. Even though the number of patients included in this study is small, it was possible to show an important modification in the content of microorganisms and resistance markers present in the microbiota. We also demonstrated the possibility of using microorganisms as biomarkers of a person’s health status or biosensors of any toxicant exposure through the study of microbial genetic markers. These genetic markers are indicative of disease progression and/or can indicate the type of metal(loid) or antibiotic to which the patient has been exposed due to genetic determinants in the microorganism of HGM. This description is particularly important in patients suffering an important decrease in one of the most important detoxification systems of the body, the liver.

## Conclusions

To our knowledge, this is the first work that shows a relationship between the presence or absence of antibiotic resistance markers, determined by qPCR, among subjects at different stages of CKD. Our results suggest modification of intestinal microbial resistome as a function of disease progression. The presence of antibiotic and metal(loid) resistance gene markers were found. *cadA* and *arsC* were detected in CKD3 and decreased gene amplification in advanced stages of CKD were observed, possibly associated with a decrease in resident HGM.

Although associated morbidities such as diabetes can result in CKD, there are other associated factors, such as metal(loid)s consumption [19]. According to recent studies, the resistome could influence the development and progression of CKDu [18], metal(loid)s exposure and the presence of antibiotic resistance genes could change over the course of CKD, thus future research is needed to advance the understanding of this relationship.

## Supplementary Information

**Additional file 1: Figure S1.** Colony count of viable bacteria (CFU/mL) of healthy and CKD3 stool samples seeded on YCFAm agar cultivated under aerobic conditions in the presence of *E. coli* MIC of arsenic or lead.

**Additional file 2: Figure S2.** Growth curves of bacteria from the microbiota of stool samples from subjects with CKD3 and healthy controls. Growth curves from bacteria of fecal samples obtained from subjects with CKD3 and healthy subjects were analyzed in YCFAm medium with and without metal(loid) supplementation of ½ MIC of *E. coli*. No significant differences were found between treatments in each group, n = 12.

**Additional file 3: Figure S3.** Measures of alpha-diversity. Observed Shannon and Simpson indexes were calculated for **A** Metal(loid)s, and **B** antibiotic resistance isolate colonies obtained from healthy and CKD3 stool samples. Indexes were calculated using the phyloseq package in RStudio, significance was calculated by ANOVA and Wilcoxon test (p = 0.3227) for the Shannon index.

**Additional file 4: Figure S4.** Pearson correlations of contingency of prospective data (chi-square test) for the total appearance of genes of metal(loid)s and antibiotics resistance among healthy controls and, stage 3, 4, and 5 CKD patients. Results were expressed by a heat map (Additional file 4: Figure S4A) and principal component analysis PCA (Additional file 4: Figure S4B).

**Additional file 5: Table S1.** Anthropometric and biochemical parameters of study participants.

**Additional file 6: Table S2.** Primers used for antibiotic and metal(loid) resistance gene determination by qPCR.

**Additional file 7: Table S3.** Minimum inhibitory concentration of isolated *E. coli* for cadmium, mercury, and arsenic under aerobic and anaerobic conditions. Assays were carried out with 3 technical repetitions and 3 biological repetitions for each metal.

## Data Availability

Not applicable.

## Abbreviations

CND: Chronic non-communicable diseases
HGM: Human gut microbiota
CKD: Chronic kidney disease
CKDu: Chronic kidney disease of unknown etiology
CKD3: Stage 3 chronic kidney disease
GFR: Glomerular filtration rate
UTI: Urinary tract infection
RRT: Renal replacement therapy
SCFA: Short-chain fatty acids
YCFAm: Modified yeast chain fatty acids medium
eGFR: Estimated glomerular filtration rate
