# Supplementary material for: Characterization of metal(loid)s and antibiotic resistance in bacteria of human gut microbiota from chronic kidney disease subjects
Source: Biol Res. 2022 Jun 17;55:23. doi: 10.1186/s40659-022-00389-z (PMC9205139; doi:10.1186/s40659-022-00389-z)
Supplement: Supplementary file 1 — Additional file 1: Figure S1. Colony count of viable bacteria (CFU/mL) of healthy and CKD3 stool samples seeded on YCFAm agar cultivated under aerobic conditions in the presence of E. coli MIC of arsenic or lead. [file 40659_2022_389_MOESM1_ESM.docx]

**A**

**Figure S1**
