# Supplementary material for: Characterization of metal(loid)s and antibiotic resistance in bacteria of human gut microbiota from chronic kidney disease subjects
Source: Biol Res. 2022 Jun 17;55:23. doi: 10.1186/s40659-022-00389-z (PMC9205139; doi:10.1186/s40659-022-00389-z)
Supplement: Supplementary file 2 — Additional file 2: Figure S2. Growth curves of bacteria from the microbiota of stool samples from subjects with CKD3 and healthy controls. Growth curves from bacteria of fecal samples obtained from subjects with CKD3 and healthy subjects were analyzed in YCFAm medium with and without metal(loid) supplementation of ½ MIC of E. coli. No significant differences were found between treatments in each group, n = 12. [file 40659_2022_389_MOESM2_ESM.docx]

A

**Figure S2**
