# Supplementary material for: Characterization of metal(loid)s and antibiotic resistance in bacteria of human gut microbiota from chronic kidney disease subjects
Source: Biol Res. 2022 Jun 17;55:23. doi: 10.1186/s40659-022-00389-z (PMC9205139; doi:10.1186/s40659-022-00389-z)
Supplement: Supplementary file 3 — Additional file 3: Figure S3. Measures of alpha-diversity. Observed Shannon and Simpson indexes were calculated for A Metal(loid)s, and B antibiotic resistance isolate colonies obtained from healthy and CKD3 stool samples. Indexes were calculated using the phyloseq package in RStudio, significance was calculated by ANOVA and Wilcoxon test (p = 0.3227) for the Shannon index. [file 40659_2022_389_MOESM3_ESM.docx]

**A**

**B**

**Figure S2**
