# Supplementary material for: Characterization of metal(loid)s and antibiotic resistance in bacteria of human gut microbiota from chronic kidney disease subjects
Source: Biol Res. 2022 Jun 17;55:23. doi: 10.1186/s40659-022-00389-z (PMC9205139; doi:10.1186/s40659-022-00389-z)
Supplement: Supplementary file 4 — Additional file 4: Figure S4. Pearson correlations of contingency of prospective data (chi-square test) for the total appearance of genes of metal(loid)s and antibiotics resistance among healthy controls and, stage 3, 4, and 5 CKD patients. Results were expressed by a heat map (Additional file 4: Figure S4A) and principal component analysis PCA (Additional file 4: Figure S4B). [file 40659_2022_389_MOESM4_ESM.docx]

**A**


**B**

**Figure S4**
