# Supplementary material for: Characterization of metal(loid)s and antibiotic resistance in bacteria of human gut microbiota from chronic kidney disease subjects
Source: Biol Res. 2022 Jun 17;55:23. doi: 10.1186/s40659-022-00389-z (PMC9205139; doi:10.1186/s40659-022-00389-z)
Supplement: Supplementary file 5 — Additional file 5: Table S1. Anthropometric and biochemical parameters of study participants. [file 40659_2022_389_MOESM5_ESM.docx]

|  | Stage | Age | Gender | Allergies | Other declared diseases | Body mass index B.M.I. | Lean Body Mass L.B.M % (Normal range Male 40-50, Female 30-40) | Percentage Body Fat P.B.F (acceptable male 25-31, female 18-25) | waist-to-hip ratio W.H.R. (Normal Range male <0.9, Female <0.8) | albuminuria (mg/24 h) | Creatinine (mg/dL) (Normal range Male 0.7 - 1.3, Female 0.6 - 1.1) | eGFR (mL/min/1.73 m2) | Urea (g/L) (normal range 0.1 - 0.5) | Glucose (mg/dL) (normal range 70 - 110) | Albumin (g/dL) (normal range 3.5 - 4.8) | Calcium (mg/dL) (Normal range 8.5 - 10.5) | Phosphorous (mg/dL) (Normal range 2.5 - 5.6) |
| --- | --- | --- | --- | --- | --- | --- | --- | --- | --- | --- | --- | --- | --- | --- | --- | --- | --- |
| Subject 1 | Healthy control | 65 | Male | No | Arterial hypertension, dyslipidemia | 24,4 | 55,1 | 25,3 | 0,96 | <30 | 0,8 | 94 | 0,24 | 85 | 4,5 | 9 | 4 |
| Subject 2 | Healthy control | 65 | Female | No | No | 25 | 41 | 23 | 0,84 | <30 | 0,84 | 73 | 0,25 | 84 | 4,4 | 9,1 | 3,7 |
| Subject 3 | Healthy control | 53 | Female | No | Arterial hypertension, dyslipidemia | 24,9 | 44,1 | 25 | 0,81 | <30 | 0,88 | 75 | 0,26 | 88,08 | 4,04 | 8,17 | 4,01 |
| Subject 4 | Healthy control | 63 | Female | No | No | 24,4 | 42 | 25 | 0,85 | <30 | 1 | 60 | 0,25 | 86,6 | 4,51 | 7,94 | 4,77 |
| Subject 5 | G3 stage CKD | 66 | Male | No | Arterial hypertension, dyslipidemia | 24,1 | 43,5 | 25,6 | 1,02 | 34,1 | 2,02 | 34 | 0,96 | 113,9 | 4,72 | 9,42 | 3,01 |
| Subject 6 | G3 stage CKD | 51 | Male | No | Arterial hypertension, Cardiovascular Disease | 25 | 50 | 20 | 0,93 | 160,25 | 2,27 | 30 | 0,99 | 103,8 | 3,89 | 9,01 | 4,15 |
| Subject 7 | G3 stage CKD | 63 | Female | No | Arterial hypertension | 24,9 | 41,2 | 31 | 0,94 | 115,37 | 2,2 | 31 | 0,5 | 67,5 | 4,05 | 10,6 | 4,85 |
| Subject 8 | G3 stage CKD | 64 | Male | No | No | 25 | 42 | 30 | 0,8 | 150 | 2 | 35 | 0,8 | 90 | 4 | 10 | 3,5 |
| Subject 9 | G4 stage CKD | 44 | Female | No | No | 23,6 | 42,9 | 28,1 | 0,82 | 72,12 | 3,21 | 17 | 0,93 | 88,6 | 3,7 | 13 | 4,72 |
| Subject 10 | G4 stage CKD | 52 | Male | No | dyslipidemia | 25,2 | 50 | 31 | 0,9 | 80 | 2,72 | 26 | 0,89 | 90,67 | 6,19 | 8,31 | 3,75 |
| Subject 11 | G4 stage CKD | 48 | Male | No | No | 24,2 | 58,8 | 22,5 | 0,93 | 105 | 3,04 | 23 | 0,9 | 93 | 4,2 | 8,7 | 3,7 |
| Subject 12 | G4 stage CKD | 58 | Female | No | No | 29,3 | 48,6 | 30 | 0,89 | 84 | 2,34 | 22 | 0,4 | 73,87 | 4,37 | 9,48 | 3,86 |
| Subject 13 | G5 stage CKD | 59 | Female | No | No | 25,1 | 34,5 | 36,3 | 0,88 | N/D | 16,09 | 2 | 92 | 92 | N/D | 10,1 | 4,7 |
| Subject 14 | G5 stage CKD | 37 | Female | No | No | 24 | 38 | 30 | 0,79 | N/D | 13,05 | 3 | 135 | 135 | N/D | 9,3 | 6,3 |
| Subject 15 | G5 stage CKD | 40 | Female | No | Arterial hypertension | 27,4 | 45,2 | 37 | 0,87 | N/D | 14,88 | 3 | 83 | 83 | N/D | 9 | 7,5 |
| Subject 16 | G5 stage CKD | 60 | Female | No | Arterial hypertension | 24,5 | 42 | 33 | 0,8 | N/D | 12 | 3 | 105 | 91 | N/D | 9,5 | 7,3 |

**Table S1**
