# Supplementary material for: Characterization of metal(loid)s and antibiotic resistance in bacteria of human gut microbiota from chronic kidney disease subjects
Source: Biol Res. 2022 Jun 17;55:23. doi: 10.1186/s40659-022-00389-z (PMC9205139; doi:10.1186/s40659-022-00389-z)
Supplement: Supplementary file 6 — Additional file 6: Table S2. Primers used for antibiotic and metal(loid) resistance gene determination by qPCR. [file 40659_2022_389_MOESM6_ESM.docx]

| **Gene** | **Product** | **Primer forward** | **Tm** | **Primer reverse** | **Tm** | **Amplicon size** | **Reference** |
| --- | --- | --- | --- | --- | --- | --- | --- |
| *catB4* | Chloramphenicol aceltitrasferase | AAGGCAAGCTGCTTTCTGAG | 55,8°C | GATAAAGGAAGCCCCACTCC | 55,2°C | 188 pb | [26] |
| *floR* | Chloramphenicol transporters | TCGTCATCTACGGCCTTTTC | 54,7°C | CTTGACTTGATCCAGAGGGC | 55,4°C | 188 pb | [26] |
| *oxa10* | Class D 𝛽-lactamase | AGAGGCTTTGGTAACGGAGG | 56,8°C | TGGATTTTCTTAGCGGCAAC | 56,5°C | 191 pb | [26] |
| *strB* | Aminoglycoside phosphotransferase | GCCTGTTTTTCCTGCTCATT | 53,8°C | CGCAGTTCATCAGCAATGTC | 54,5°C | 150 pb | [26] |
| *qnrB1* | Fluoroquinolone | AAATATGGCTCTGGCACTCG | 55,1°C | CTTTCAGCATCGCACGACTA | 55,1°C | 191 pb | [26] |
| *mphB* | Macrolide 2 'phosphotransferase | TGTGCCAGCAGGTACGATAG | 56,8°C | CCATACGCTGCTTCATTGAC | 54,1°C | 100 pb | [26] |
| *ermB* | rRNA adenine N6 -methyltransferase | AGCCATGCGTCTGACATCTA | 56,0°C | CTGTGGTATGGCGGGTAAGT | 56,9°C | 193 pb | [26] |
| *mefE1* | MFS efflux proteins | CCTGCAAATGGCGATTATTT | 51,6°C | AATAGCAAGCACTGCACCAG | 56,9°C | 199 pb | [26] |
| *arr2* | Rifampicin ADP-ribosylation transferase | TTACAAGCAGGTGCAAGGAC | 55,1°C | GCTCCATCAAGGCTGAAAAG | 54,1°C | 140 pb | [26] |
| *sulll* | Sulfonamides dihydropteroate synthetase | GACAGTTATCAACCCGCGAC | 55,9°C | GTCTTGCACCGAATGCATAA | 54,1°C | 147 pb | [26] |
| *dhfr1* | Trimethoprim dihydrofolate reductase | ATGGAGTGCCAAAGGTGAAC | 55,6°C | TGGAAAGATCACTACGTTCTCA | 53,3°C | 161 pb | [26] |
| *tetA* | Tetracycline transporter | TTGCTTCGGAAGATATCGCT | 54,0°C | ATCCAAAGCGCACTTGAAAA | 53,4°C | 200 pb | [26] |
| *acrB* | Multi-Drug Outflow Pumps | ATATCCTACGATTGCACCGC | 54,9°C | GGTACCCGTGGAGTCACTGT | 58,8°C | 160 pb | [26] |
| *arsA* | Arsenic ATPase pump | CGAAACAAACGAAAGTAATTGTTG | 51,7°C | ATGCTTCCTTTAGAAAAGGAACAA | 53,0°C | 252 pb | This work |
| *arsC* | Arsenate reductase | ACAGAACCGACCGTTATTCATTAT | 54,2°C | TCCCAGCGGCGTCACCACAATCGG | 67,5°C | 228 pb | This work |
| *cadA2k* | Cadmium translocator P-type ATPase | GCTGAGGTAACTGACATATTGGTG | 55,5°C | CACCATCTATCAGTGTGACTGTCT | 56,0°C | 295 pb | This work |
| *cadA3k* | Cadmium translocator P-type ATPase | GTAGATTATGCGGTGGTTTCTTTT | 53,6°C | CCCGATGTACATCAGGATCAAAAA | 55,1°C | 241 pb | This work |
| *merA* | Mercury ion reductase | CACTCGTGCCACTATCACCA | 57,0°C | TTGTCGAACTCGGCCAGCACGCGC | 68,1°C | 195 pb | This work |
| *pbrA* | Membrane-anchored heavy metal resistance ATPase | GGCACAGATACCGCTATTGAA | 54,9°C | CATCCACATGGTCGCGTGACCCGG | 66,0°C | 241 pb | This work |

**Table S2**
