# Supplementary material for: Characterization of metal(loid)s and antibiotic resistance in bacteria of human gut microbiota from chronic kidney disease subjects
Source: Biol Res. 2022 Jun 17;55:23. doi: 10.1186/s40659-022-00389-z (PMC9205139; doi:10.1186/s40659-022-00389-z)
Supplement: Supplementary file 7 — Additional file 7: Table S3. Minimum inhibitory concentration of isolated E. coli for cadmium, mercury, and arsenic under aerobic and anaerobic conditions. Assays were carried out with 3 technical repetitions and 3 biological repetitions for each metal. [file 40659_2022_389_MOESM7_ESM.docx]

| Metal(oid) | Aerobic (mM) | Anaerobic (mM) |
| --- | --- | --- |
| Cadmium | 1 (12) | 1 (12) |
| Mercury | 0.01 (12) | 0.001 (12) |
| Arsenic | 10 (12) | 1 (12) |
| Lead | 0.001 (12) | 0.001 (12) |

**Table S3**
